# Essential Trace Elements Zinc, Iron, Copper and Attention-Deficit/Hyperactivity Disorder in Children and Adolescents: A Systematic Review and Meta-Analysis of Case–Control Studies

**DOI:** 10.3390/nu18111797

**Published:** 2026-06-02

**Authors:** Wei Wang, Lei Tian, Huiqiong Xu, Jixing Zhou, Menglong Geng

**Affiliations:** 1 Department of Public Health, Suzhou Vocational Health College, 28 Kehua Road, Suzhou 215000, China; wangwei@szhct.edu.cn; 2 Anji County Center for Disease Control and Prevention, No 286 Shifo Road, Huzhou 313000, China; mj1999728@dingtalk.com; 3 School of Public Health and Health Management, Anhui Institute of Medicine, No. 632 Furong Road, Heifei 230032, China; xhqxuhuiqiong@163.com; 4 School of Public Health, Southwest Medical University, No. 1, Section 1, Xianglin Road, Longmatan District, Luzhou 646000, China; 5 Key Laboratory of Population Health Across Life Cycle, Anhui Medical University, Ministry of Education of the People’s Republic of China, No. 81 Meishan Road, Hefei 230032, China; 6 Anhui Provincial Key Laboratory of Environment and Population Health Across the Life Course, Anhui Medical University, No. 81 Meishan Road, Hefei 230032, China

**Keywords:** zinc, iron, copper, ADHD, meta-analysis

## Abstract

Essential trace elements such as zinc (Zn), iron (Fe), and copper (Cu) play a critical role in neurodevelopment, influencing key processes like neurotransmitter regulation and neuronal signaling. To synthesize the existing evidence, we conducted a systematic review and meta-analysis of case–control studies investigating the associations between these trace elements and Attention-Deficit/Hyperactivity Disorder (ADHD) in children and adolescents. A comprehensive literature search was performed up to March 2026 across multiple databases, including Web of Science, PubMed, and the Chinese National Knowledge Infrastructure. A total of 46 studies involving 5515 ADHD cases and 8166 controls were included. The results showed that Zn (SMD = −1.01, 95% CI: −1.51 to −0.52), Fe (SMD = −0.82, 95% CI: −1.52 to −0.11) and Ferritin (SMD = −0.54, 95% CI: −1.00 to −0.27) levels were significantly lower in children with ADHD than in controls, while no significant difference was observed for Cu levels (SMD = −0.55, 95% CI: −1.12 to 0.02). When the research subjects are limited to children ≤12 years old, the differences in Zn are more pronounced. Moreover, the differences in the levels of Zn, Fe, and Cu were more pronounced among children and adolescents from developing countries. These findings suggest that imbalances in Zn and Fe may be involved in the pathogenesis of ADHD. Further research is needed to validate early biomarkers of ADHD risk and to explore their potential application in clinical diagnosis and management, especially considering the high heterogeneity of the studies included in this study.

## 1. Introduction

Attention-Deficit/Hyperactivity Disorder (ADHD) is a neurodevelopmental disorder primarily characterized by developmentally inappropriate, persistent, and impairing levels of inattention, hyperactivity, and impulsivity, which stem from widespread yet subtle alterations in gene expression across multiple brain regions that collectively disrupt typical neural functioning [1,2]. It is typically diagnosed in childhood and often persists into adulthood. Recent data report ADHD prevalence rates of 7.6% among children aged 3–12 years and 5.6% among adolescents aged 12–18 years [3]. Between 2016 and 2019, nearly one in ten (9.8%) children in the United States received an ADHD diagnosis based on parent-reported data [4]. The prevalence of ADHD among Chinese children and adolescents in China has been estimated at 6.3% [5]. Children and adolescents with ADHD often experience psychosocial difficulties across home, school, and community settings [6], which may increase their risk of entering a negative developmental trajectory.

There is ample evidence for the heritability of ADHD, but environmental factors, including their direct effects and interactions with genes, play a crucial role in determining the severity and clinical characteristics of symptoms, which in turn affect diagnosis and treatment pathways [7,8]. Given the crucial role of micronutrients in neurodevelopment, nutritional impairment has been closely associated with ADHD [9]. Trace elements such as zinc (Zn), copper (Cu), and iron (Fe) play essential roles in normal brain development, neurotransmitter synthesis and catabolism, cellular metabolism, and dopaminergic pathways [10,11]. Altered levels of these elements may disrupt the regulation of dopaminergic and noradrenergic systems, potentially contributing to the pathogenesis of ADHD [12].

Nevertheless, existing epidemiological studies—particularly case–control designs—on the association between Zn, Cu, and Fe levels and ADHD have yielded inconsistent and sometimes contradictory results. Some studies report deficiencies of Zn [13,14,15,16,17], Fe [13,14], and Cu [18,19] in children with ADHD, while others describe elevated serum levels of Zn [20,21], Cu [22,23,24,25], and Fe [26] in affected individuals. These discrepancies may stem from variations in biological samples, participant age ranges, and population representativeness. For instance, one study observed lower blood Zn levels in both the 6–11 and 12–16 age groups of children with ADHD compared to controls, whereas Fe levels were significantly lower only in the 6–11 age group, and Cu levels were reduced solely in the 12–16 age group [15]. Furthermore, studies have suggested that the Cu-to-Zn ratio in children’s urine or hair may serve as a precise biomarker of ADHD etiology, aiding in both identification and monitoring [22].

Considering the various side effects of conventional pharmacotherapy for ADHD [27], researchers have begun to explore novel therapeutic approaches, particularly the investigation of the potential benefits of mineral supplementation [28]. Recent systematic reviews indicated that the specific role of dietary nutrients, including Zn and Fe, in treating ADHD remains controversial, although evidence supporting Zn supplementation appears to be the most robust [29,30]. Particularly in regions with inadequate diet and nutritional deficiencies, early exposure to micronutrient supplementation may yield more pronounced benefits on children’s behavioral outcomes [31].

Although research in this field has increased in recent years, the association between levels of Zn, Fe, and Cu and the onset of ADHD in children and adolescents remains unclear. There is an urgent need to comprehensively analyze the existing literature to establish an evidence-based foundation for early intervention and prevention strategies for ADHD. Therefore, this study systematically reviews the existing evidence regarding the relationship between Zn, Fe, and Cu and ADHD in children and adolescents.

## 2. Methods

The study followed the PRISMA and MOOSE guidelines for conducting and reporting the meta-analysis.

### 2.1. Literature Search

Two investigators (Wei Wang and Lei Tian) independently conducted a comprehensive literature search across PubMed, Web of Science, and the China National Knowledge Infrastructure (CNKI) up to March 2026. An example search strategy was as follows: (‘ADHD’ OR ‘Attention-Deficit/Hyperactivity Disorder’ OR ‘Hyperkinetic Disorder’ OR ‘Conduct Disorder’) AND (‘Zn’ OR ‘Fe’ OR ‘Cu’ OR ‘Zinc’ OR ‘Iron’ OR ‘Copper’ OR ‘Trace Elements’ OR ‘metal’) AND (‘child’ OR ‘adolescent’ OR ‘Teenagers’ OR ‘youth’ OR ‘juvenile’ OR ‘Infant’ OR ‘Young Adult’). Inclusion criteria comprised human studies published in English or Chinese, with no restriction on publication date. The complete search strategy is provided in Table S1.

The search was first performed in each database using the formulated strategy. Retrieved records were imported into EndNote X8 for duplicate removal. Titles and abstracts were then screened to identify potentially relevant studies. If both reviewers considered an abstract relevant, the full text was obtained and independently assessed. Additionally, reference lists of included articles were manually examined to identify further eligible studies.

### 2.2. Study Selection

Studies investigating the association between essential trace elements and ADHD in children and adolescents were included. The selection criteria were as follows: (1) Study Design: Case–control studies. (2) Participants: Children and adolescents. (3) Exposure: Levels of Zn, Fe, and/or Cu. (4) Outcome: ADHD diagnosis compared with non-ADHD controls. (5) Data Required: Means and standard deviations (SD) of metal concentrations for both ADHD and healthy control groups. (6) Language: Published in English or Chinese. Review articles, editorials, and animal studies were excluded. When multiple studies reported identical data, the study with the larger sample size was selected for inclusion in the meta-analysis.

### 2.3. Quality Assessment

The Newcastle-Ottawa Scale (NOS) for case–control studies was used to assess the quality of included studies. Two researchers (Wei Wang and Lei Tian) independently performed the quality evaluation, with any disagreements resolved through discussion or by consultation with a third researcher (Menglong Geng).

### 2.4. Data Extraction and Analysis

The following information was extracted from each included study: first author, publication year, country, participant age, sample size, study design, sex, biological samples, and metal levels.

All statistical analyses were conducted using STATA (version 15.0). A *p*-value < 0.05 was considered statistically significant. For studies reporting mean metal concentrations in blood, urine, or hair of children with and without ADHD, a pooled mean difference with its 95% confidence interval was computed. Stratified analyses were then performed according to biological sample type, geographic region, age, and sample size.

Results are presented in forest plots or tables. Heterogeneity among studies was quantified using the I^2^ statistic. An I^2^ > 50% was considered indicative of substantial heterogeneity, in which case a random-effects model was applied; otherwise, a fixed-effects model was used.

Sensitivity analysis was performed by sequentially removing each included study to examine its influence on the overall mean difference. Stability of the results was assumed if the pooled estimates remained largely unchanged after exclusion of any single study. Publication bias was evaluated visually via funnel-plot symmetry and statistically with Egger’s test. When evidence of bias was detected, the trim-and-fill method was applied to recalculate the pooled effect. Using the Galbraith plot for heterogeneity assessment and visualization. Meta-regression was conducted to explore potential sources of heterogeneity (including publication year, biological sample type, region, and sample size).

## 3. Results

### 3.1. Search Results

**Figure 1:**
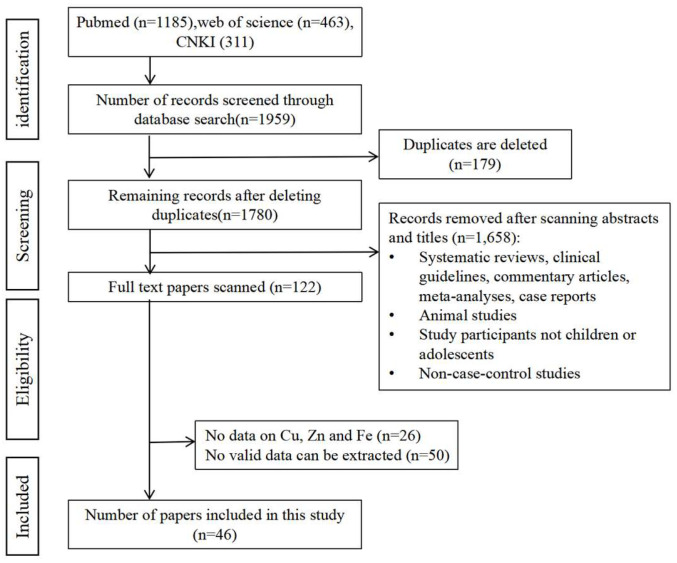
Flow chart for article selection.

A total of 1959 citations were retrieved from four databases. After removing 179 duplicates, the titles and abstracts of the remaining 1780 records were screened, resulting in 122 studies selected for full-text review. Following further exclusions, 46 studies were ultimately included in the final meta-analysis (Figure 1).

### 3.2. Characteristics of Included Studies

The characteristics of the 46 included studies are presented in Table 1. Geographically, the studies were predominantly from Asia (*n* = 28) and Europe (*n* = 10), with 8 each from Africa, Oceania, and the Americas. All studies received quality scores above 6, with an average score of 6.9. Among the included publications, Zn levels were reported in 36 studies (37 datasets), Fe levels in 23 studies (23 datasets), ferritin levels in 9 studies (9 datasets), and Cu levels in 24 studies (24 datasets). Most studies measured metals in blood (serum, plasma, or whole blood; *n* = 38), followed by hair (*n* = 6) and urine (*n* = 2). The largest number of studies (*n* = 30) were published between 2010 and 2025. The vast majority of studies included both boys and girls (*n* = 44).

### 3.3. Findings from the Studies

#### 3.3.1. Meta-Analysis of Mean Zn Levels

**Figure 2:**
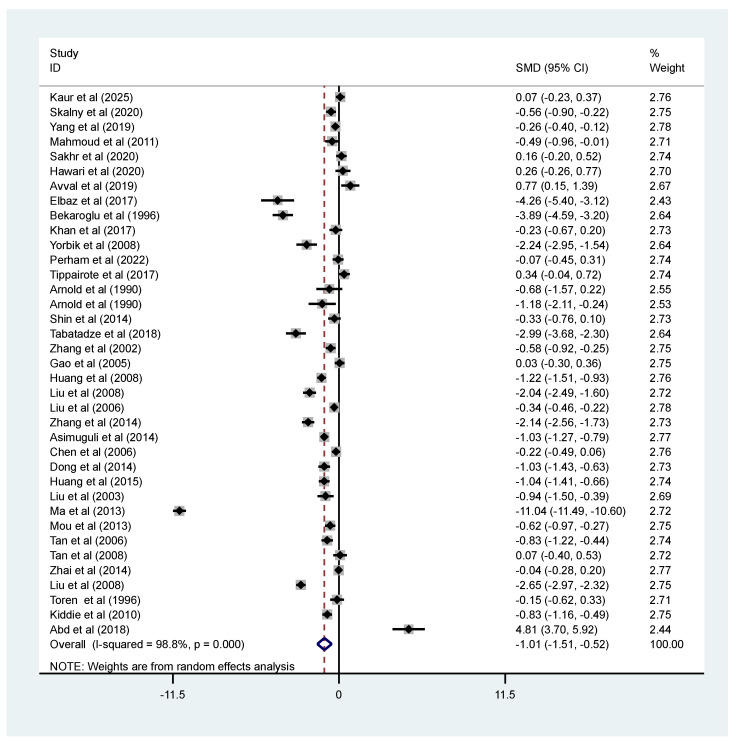
Forest plot illustrating the association between Zn levels and ADHD in children and adolescents, presented as the mean difference between case and control groups. Diamonds indicate pooled estimates derived from random-effects meta-analysis. Abbreviation: SMD, standardized mean difference [14,15,17,19,20,21,25,32,33,34,35,36,37,38,39,40,41,42,43,44,45,46,47,48,49,50,51,52,53,54,55,56,57,59,60,67].

Regarding Zn levels, this meta-analysis included a total of 36 studies. Compared with healthy controls, children and adolescents with ADHD had significantly lower Zn levels (SMD = −1.01, 95% CI: −1.51 to −0.52). High heterogeneity was observed among studies (I^2^= 98.8%, *p* < 0.001), and therefore a random-effects model was used for the pooled analysis (Figure 2).

Subgroup analyses by biological sample type showed significantly lower Zn levels in blood samples (SMD = −1.09, 95% CI: −1.66 to −0.52) from ADHD cases, but no significant differences in urine (SMD = −0.47, 95% CI: −1.68 to 0.74) or hair (SMD = −0.71, 95% CI: −1.65 to 0.22). Further stratified by sample size, the inverse association persisted across all subgroups: sample size <100 (SMD = −0.77, 95% CI: −1.51 to −0.02), 100–200 (SMD = −0.63, 95% CI: −1.03 to −0.24), and >200 (SMD = −1.95, 95% CI: −3.21 to −0.69), indicating robustness of the finding irrespective of study scale. The pattern also remained consistent in analyses restricted to participants aged <12 years (SMD = −1.25, 95% CI: −2.05 to −0.46). Stratified by economic level, studies in developing countries (SMD = −1.14, 95% CI: −1.73 to −0.58) showed a more significant decrease in Zn levels in ADHD cases compared to studies in developed countries (SMD = −0.37, 95% CI: 0.70 to −0.04) (Table 2).

#### 3.3.2. Meta-Analysis of Mean Fe and Ferritin Levels

**Figure 3:**
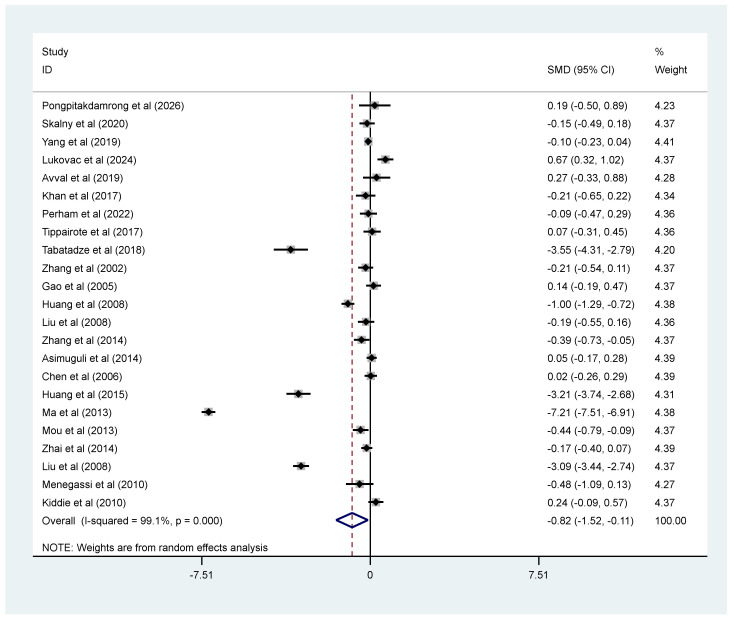
Forest plot illustrating the association between Fe levels and ADHD in children and adolescents, presented as the mean difference between case and control groups. Diamonds indicate pooled estimates derived from random-effects meta-analysis. Abbreviation: SMD, standardized mean difference [14,15,17,18,20,21,26,36,38,41,42,43,44,46,47,48,50,52,53,56,57,59,64].

Regarding Fe levels, this meta-analysis included a total of 23 studies. Compared with healthy controls, children and adolescents with ADHD exhibited significantly lower Fe levels (SMD = −0.82, 95% CI: −1.52 to −0.11). High heterogeneity was present among the studies (I^2^ = 99.1%, *p* < 0.001), warranting the use of a random-effects model (Figure 3).

**Figure 4:**
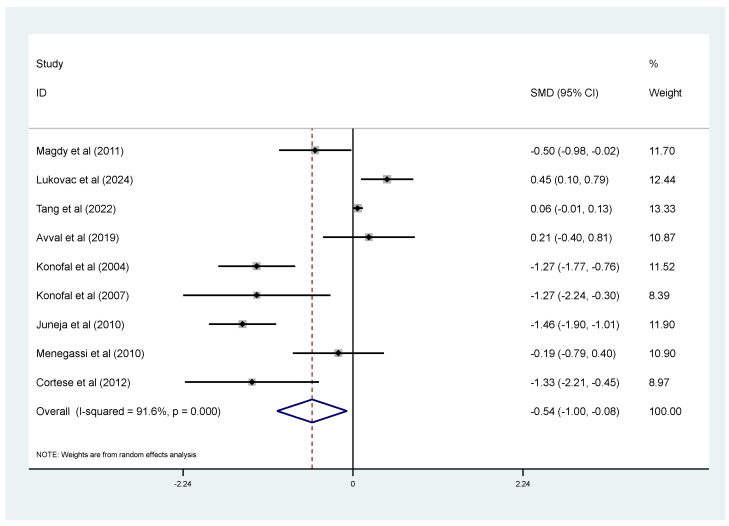
Forest plot illustrating the association between ferritin levels and ADHD in children and adolescents, presented as the mean difference between case and control groups. Diamonds indicate pooled estimates derived from random-effects meta-analysis. Abbreviation: SMD, standardized mean difference [21,26,32,58,61,62,63,64,65].

Regarding ferritin levels, this meta-analysis included a total of 9 studies. Compared with healthy controls, children and adolescents with ADHD exhibited significantly lower Fe levels (SMD = −0.54, 95% CI: −1.00 to −0.08). Substantial heterogeneity was present among the studies (I^2^ = 91.6%, *p* < 0.001), warranting the use of a random-effects model (Figure 4).

Subgroup analysis by biological sample type showed significantly lower Fe levels in blood samples (SMD = −0.81, 95% CI: −1.61 to −0.02) of ADHD cases, whereas no significant difference was observed in hair samples (SMD = −0.81, 95% CI: −2.10 to 0.47). In the analysis restricted to participants aged <12 years, the difference approached but did not reach statistical significance (SMD = −0.66, 95% CI: −1.48 to 0.15). According to the analysis of economic level, the decrease in Fe level is only statistically significant in studies conducted in developing countries (SMD = −0.98, 95% CI: −1.77 to −0.19), and no significant difference was observed in studies conducted in developed countries (SMD = −0.28, 95% CI: 0.82 to 0.26) (Table 2).

For ferritin, due to limited inclusion in the study, we did not conduct additional subgroup analysis.

#### 3.3.3. Meta-Analysis of Mean Cu Levels

**Figure 5:**
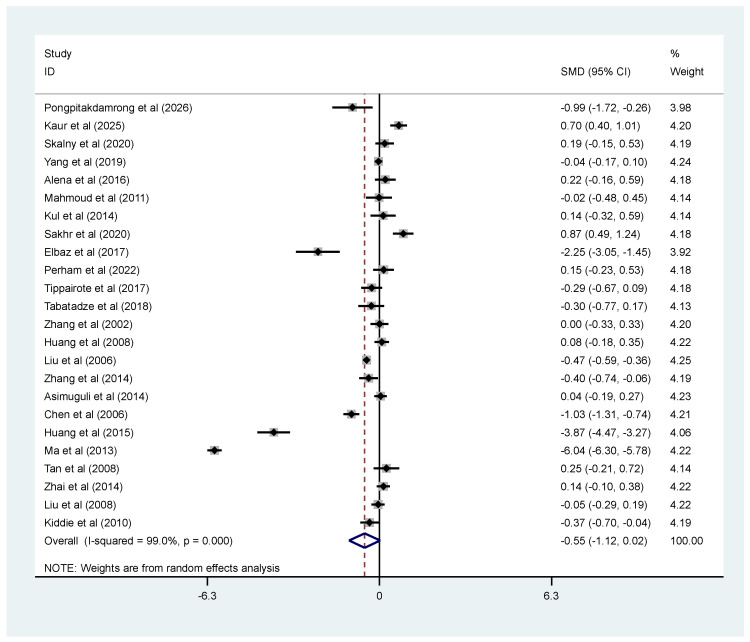
Forest plot illustrating the association between Cu levels and ADHD, presented as the mean difference between case and control groups. Diamonds indicate pooled estimates derived from random-effects meta-analysis. Abbreviation: SMD, standardized mean difference [14,15,16,17,18,19,20,25,32,33,38,41,43,45,46,47,48,50,52,55,56,57,59,66].

Regarding Cu levels, this meta-analysis included a total of 24 studies. Compared with healthy controls, children and adolescents with ADHD showed lower Cu levels, though the difference did not reach statistical significance (SMD = −0.55, 95% CI: −1.12 to 0.02). High heterogeneity was observed across studies (I^2^ = 99.0%, *p* < 0.001), leading to the use of a random-effects model (Figure 5).

Subgroup analysis by biological sample type indicated lower Cu levels in blood (SMD = −0.66, 95% CI: −1.32 to 0.01) and hair samples (SMD = −0.28, 95% CI: −0.66 to 0.11) of ADHD participants, but neither difference was statistically significant. When stratified by sample size, lower Cu levels were observed across all subgroups: <100 (SMD = −0.46, 95% CI: −1.05 to 0.13), 100–200 (SMD = −0.24, 95% CI: −0.87 to 0.38), and >200 (SMD = −0.86, 95% CI: −1.91 to 0.20). None of these differences reached statistical significance. In the analysis limited to participants aged <12 years, the difference remained statistically non-significant (SMD = −0.66, 95% CI: −1.58 to 0.27). Geographic subgroup analysis showed a significant reduction in Cu levels only in studies conducted in developing countries (SMD = −0.74, 95% CI: −1.41 to −0.06), whereas no significant difference was found in studies from developed nations (SMD = 0.17, 95% CI: −0.21 to 0.55) (Table 2).

### 3.4. Sensitivity Analyses and Publication Bias Diagnostics

The sensitivity analysis showed that the pooled SMD did not exceed the original confidence interval after removing each study and indicated that the result was stable. (Figure S1).

In the meta-analyses comparing metal levels between children with and without ADHD, no evidence of publication bias was detected based on funnel plots or Egger’s test results [Zn: *p* = 0.080; Fe: *p* = 0.105; ferritin: *p* = 0.069; Cu: *p* = 0.792] (Figure S2). However, Begg’s test suggested potential publication bias for Fe (*p* = 0.021) and Cu (*p* = 0.031) comparisons (Table S2).

### 3.5. Heterogeneity Test, Meta-Regression and Trim-And-Fill Analysis

Heterogeneity analysis revealed high heterogeneity in the pooled differences in Zn, Fe, ferritin and Cu levels between children and adolescents with ADHD and control groups in this study (Figure S3).

The results of meta regression suggest that differences in publication year, biological sample, region, and sample size are not sufficient to explain the main sources of high heterogeneity (Table S4).

To assess the robustness of the findings against potential publication bias, a trim-and-fill analysis was conducted. The adjusted results indicated only minor changes in the effect sizes for Zn (SMD = −1.67; 95% CI: −2.17 to −1.16), Fe (SMD = −1.13; 95% CI: −1.77 to −0.48), while ferritin showed no significant change (SMD = −0.54; 95% CI: −1.00 to −0.08), supporting the stability of the pooled estimates (Figure S4). Notably, after imputation, the association for Cu became statistically significant (SMD = −0.92; 95% CI: −1.44 to −0.41) (Figure S4) (Table S3).

## 4. Discussion

Based on a meta-analysis of 46 studies, we found that children and adolescents with ADHD had significantly lower levels of Zn, Fe and ferritin compared to the control group, suggesting that deficiencies in these micronutrients may be associated with the occurrence of ADHD. In addition, the Cu levels in children and adolescents with ADHD were lower than those in the control group, but the difference was not statistically significant. While earlier meta-analyses also observed reduced Zn levels in children with ADHD, the pooled effects did not reach statistical significance [68,69]. The current analysis, which includes a larger number of studies and a greater total sample size, has substantially enhanced statistical power and result reliability, increasing the likelihood of detecting true intergroup differences. Moreover, this is the first meta-analysis to quantitatively assess Fe, ferritin and Cu levels in children and adolescents with ADHD.

Zn deficiency may participate in the pathological process of ADHD by disrupting neurotransmitter metabolism, such as dopamine synthesis and signaling, [70] and impairing synaptic plasticity [71]. Fe deficiency may disrupt neurotransmitter balance, reduce myelination, impair synaptogenesis, and affect basal ganglia function [72,73]. Deficiencies in both Zn and Fe might jointly influence critical neurodevelopmental pathways [74]. However, the precise underlying mechanisms remain unclear and warrant further investigation.

Our study identified significantly lower Zn and Fe levels in children and adolescents with ADHD relative to controls, albeit with considerable heterogeneity across studies, leading us to apply random-effects meta-analysis models. Restricting the analysis to participants aged 12 years or younger yielded effect estimates consistent in direction and magnitude with the primary findings. In the subgroup analysis of Fe metabolism indicators, we conducted subgroup analysis based on indicator types (serum Fe and ferritin). The results showed that the serum Fe and ferritin levels in children with ADHD were significantly lower than those in healthy control children, suggesting that there may be a common situation of decreased Fe reserves and insufficient circulating Fe supply in ADHD children and adolescents. Fe deficiency may participate in the pathophysiology of ADHD by disrupting dopamine synthesis, transport, and receptor function, as well as affecting neural activity in key brain regions such as the basal ganglia [75,76]. Subgroup analysis based on biological sample type revealed that differences in Zn and Fe levels were more pronounced in blood samples compared to other specimen types. This supports the continued use of blood-based measures for detecting and monitoring metal concentrations in clinical and epidemiological settings, although plasma-based methods are receiving increasing attention [77].

Subgroup analysis by sample size indicated that larger studies (*n* > 200) demonstrated more marked differences in Zn and Fe levels, reinforcing the main findings and implying that earlier smaller-scale studies may have underestimated the association between these micronutrients and ADHD due to limited statistical power. Further stratification by economic development level showed that Zn and Fe differences were more pronounced in developing countries, with Fe deficiency reaching statistical significance only in this subgroup. Potential factors contributing to this pattern in developing regions may include less varied diets, inadequate nutrient intake, or environmental exposures [78,79]. The prevalence of Zn deficiency in low- and middle-income countries is worrying. Globally, Africa has the highest rate of Zn deficiency (23.9%) [80], and accounts for 18 of the top 20 countries most severely affected by multiple micronutrient deficiencies, including Zn and Fe [81]. However, effective strategies to improve Zn nutrition status, such as Zn strengthening programs, micronutrient powder supplementation, dietary diversification, and meat intake promotion, still lack sufficient implementation experience and effectiveness evaluation evidence [82,83]. Additionally, in many developing countries and regions, heavy metal pollution tends to be more widespread and severe [84]. The combined effects of long-term, low-dose exposure to heavy metals such as Pb and Cd, coupled with insufficient levels of essential nutrients, are progressively causing subclinical neurological damage [85]. This emerging threat is increasingly recognized as a serious public health challenge in developing nations. These findings also suggest that monitoring and timely supplementation of Zn/Fe could represent a cost-effective intervention in resource-limited settings, particularly in developing countries [86].

Admittedly, this study only evaluated differences in trace element levels between children/adolescents with ADHD and controls using a case–control design, which cannot establish causality. Nonetheless, the results imply that Fe supplementation could be a useful alternative therapy for ADHD patients with Fe deficiency, especially those presenting predominantly inattentive symptoms [87]. A systematic review of randomized controlled trials indicated that dietary supplementation with Zn and Fe was associated with improvement in ADHD severity at the end of treatment compared with placebo, although effect sizes were generally modest and mostly limited to specific symptoms or assessment tools [29].

We also observed lower Cu levels in children with ADHD relative to controls, though the pooled effect size did not attain statistical significance. Cu is an essential trace element that acts as a cofactor for multiple oxidoreductases and is involved in Fe metabolism, antioxidant defense, neuropeptide synthesis, and immune regulation. However, reference ranges for Cu remain uncertain, especially given that excess Cu can promote oxidative stress and cytotoxicity, primarily harming the liver and nervous system, and may disturb the balance of other essential minerals [88]. Subgroup analyses stratified by region and sample type revealed that in developing countries, Cu levels were significantly lower in children with ADHD than in typically developing peers; in blood specimens, the difference in Cu levels appeared more pronounced, yet it still lacked statistical significance.

Sensitivity analyses confirmed the robustness of the main findings. Trim-and-fill analysis further validated the results for Zn and Fe, while revealing a statistically significant reduction in Cu levels among children and adolescents with ADHD compared to controls.

However, we have not fully addressed interactions among different metals or adequately controlled for potential confounding from exposure to other heavy metals and prenatal metal exposure [89,90]. For instance, some studies report that the Cu-to-Zn ratio predicts ADHD symptoms better than either metal alone [17,22]; moreover, under conditions of high exposure to toxic metals such as cadmium (Cd), arsenic (As), and mercury (Hg), low Zn levels become a stronger predictor of neurodevelopmental abnormalities [91].

Research indicates that the content of Zn and Cu in hair is positively correlated with age [20]. We further conducted an additional meta-analysis of studies including children under 12 years of age. The results showed that, compared with the control group, children with ADHD exhibited a more significant difference in Zn levels (SMD = −1.49), while the differences in Fe (SMD = −0.56) and Cu (SMD = −0.66) levels were relatively moderate. However, only a limited number of studies have directly compared Zn, Fe, and Cu levels between children with ADHD and controls across preschool age, childhood, and adolescence, and the findings have been inconsistent [13,15,17]. Most existing studies have not analyzed the groups of children (aged ≤ 12 years) and adolescents (aged > 12 years) independently. Instead, they have included both groups together. Although our subgroup analysis showed that the current main conclusions remained stable and significant in children aged ≤ 12 years, due to the wide age range of the study population included, the current results still exhibited high heterogeneity.

The meta-regression indicated that common covariates such as publication year, biological sample type, region, and sample size were insufficient in explaining the primary sources of this high heterogeneity. This suggests that the discrepancies across studies may stem from deeper clinical and methodological factors, including: inconsistent diagnostic criteria (e.g., varying versions of DSM or ICD), which may have led to differences in the clinical profiles of enrolled ADHD patients; variability in analytical methods and quality control procedures, as differences in instrumentation (e.g., ICP-MS, AAS) and sample pre-treatment across laboratories could impact the comparability of trace element concentrations; and insufficient control of key confounders, such as medication use, nutritional status, and inflammatory markers, which were often inadequately reported or adjusted for in the original studies. Consequently, although the pooled effect size reached statistical significance, its interpretation requires considerable caution. Future research should aim to advance standardization in diagnostic criteria, harmonization of analytical methods, and systematic reporting of confounders to enhance the consistency and reliability of evidence in this field.

The meta-regression indicated that common covariates such as publication year, biological sample type, region, and sample size were insufficient in explaining the primary sources of this high heterogeneity. This suggests that the discrepancies across studies may stem from deeper clinical and methodological factors. These factors include inconsistent diagnostic criteria (e.g., varying versions of DSM or ICD), which may have led to differences in the clinical profiles of enrolled ADHD patients [92]. Another important factor is variability in analytical methods and quality control procedures; for instance, differences in instrumentation (e.g., inductively coupled plasma mass spectroscopy, atomic absorption spectroscopy) and sample pre-treatment across laboratories could impact the comparability of trace element concentrations [93]. Furthermore, insufficient control of key confounders, such as medication use, nutritional status, and inflammatory markers, was often observed, as these were inadequately reported or adjusted for in the original studies [94,95,96]. Consequently, although the pooled effect size reached statistical significance, its clinical interpretation requires considerable caution. Future research should aim to advance standardization in diagnostic criteria, harmonization of analytical methods, and systematic reporting of confounders to enhance the consistency and reliability of evidence in this field.

This study also has the following limitations. First, the current findings regarding differences in Zn and Fe levels are primarily based on case–control studies, a study design that inherently struggles to establish clear causal relationships. Therefore, the observed associations may be confounded by confounding factors or reverse causality; future prospective cohort studies or randomized controlled trials are needed to further validate the direction of causality. Second, due to the lack of sufficient gender-specific data, we were unable to conduct an in-depth analysis of differences in trace element levels among children of different genders and their specific associations with ADHD risk. This limitation is particularly significant, as studies have found that among children, the proportion of males diagnosed with ADHD is significantly higher than that of females [97]. Third, it should be noted that the included studies predominantly involved populations from Asia and Europe, with a significant proportion (19 studies) conducted in China. Additionally, many of these investigations utilized relatively small sample sizes. These factors may limit the statistical power and generalizability of the findings. Therefore, further validation through larger-scale studies and the inclusion of populations from more diverse geographical regions is warranted. Fourth, another limitation of this study is that the included literature spans a long period (1996–2025), during which the diagnostic criteria for ADHD were not uniform. Notably, major revisions to ADHD diagnostic standards (e.g., from DSM-3 to DSM-5) were implemented in this period, which may have led to heterogeneity in how participants were diagnosed across studies. Finally, many of the included studies did not account for participants’ dietary intake of Zn, Fe, and Cu, which may affect the results since the intake of these trace elements is closely related to their serum concentrations [98]. Future research should concurrently assess internal exposure levels of multiple essential and toxic metals, their mutual ratios, and possible interactive effects to more precisely clarify the causal relationship between metal imbalances and ADHD.

## 5. Conclusions

In summary, this meta-analysis indicates that children and adolescents with ADHD exhibit significantly lower Zn, Fe, and ferritin levels than healthy control group. This difference is more pronounced among populations in developing countries. When the research subjects are limited to children under 12 years old, the differences in Zn are more pronounced. Reduced serum concentrations of these trace elements may be associated with ADHD. However, considering the relatively high heterogeneity of the pooled results in this study, the clinical value of the research findings needs to be carefully evaluated. Future well-controlled clinical trials are warranted to evaluate the efficacy of mineral supplementation and to identify potential subgroups of patients who may benefit.

## Figures and Tables

**Table 1:** Characteristics of selected studies.

First Author	Year of Publication	Country	Region	Age Range (Years)	Sex	Biological Samples	Sample Size	Metal Levels		NOS
								Case	Control	
Tabatadze et al. 2018 [14]	2018	Georgia	Asia	Total: 6–8	Boys and girls	Hair	Case: 35 Control: 35	Zn: 76.64 ± 16.4 Cu: 17.24 ± 5.9 Fe: 10.15 ± 3.4	Zn: 142.94 ± 26.7 Cu: 18.86 ± 4.9 Fe: 26.16 ± 5.4	8
Yang et al. 2019 [15]	2019	China	Asia	Case: 8.8 ± 2.1; Control: 8.9 ± 1.7	Boys and girls	Blood	Case: 419 Control: 395	Zn: 84.79 ± 12.46 Cu: 22.44 ± 5.39 Fe: 8.15 ± 0.8	Zn: 88.03 ± 12.38 Cu: 22.64 ± 5.64 Fe: 8.23 ± 0.87	9
Viktorinova et al. 2016 [16]	2016	Slovakia	Europe	Total: 6–14	Boys and girls	Blood	Case: 58 Control: 50	Cu: 18.31 ± 3.34	Cu: 17.64 ± 2.83	8
Skalny et al. 2020 [17]	2020	Russia	Europe	Total: 4–9	Boys and girls	Blood	Case: 68 Control: 68	Zn: 0.93 ± 0.101 Cu: 1.22 ± 0.23 Fe: 1.24 ± 0.47	Zn: 1.007 ± 0.166 Cu: 1.18 ± 0.19 Fe: 1.31 ± 0.45	7
Pongpitakdamrong et al. 2025 [18]	2026	Thailand	Asia	Total: 6–18	Boys and girls	Hair	Case: 24 Control: 12	Cu: 7.53 4.26 Fe: 10.6 ± 5.8	Cu: 21.98 ± 24.99 Fe: 9.16 ± 10.09	6
Elbaz et al. 2017 [19]	2017	Egypt	Africa	Total: 7.74 ± 1.48	Boys and girls	Blood	Case: 20 Control: 20	Zn: 69.68 ± 22.9 Cu: 92.52 ± 57.54	Zn: 159.54 ± 19.16 Cu: 189.89 ± 20.64	6
Tippairote et al. 2017 [20]	2017	Thailand	Asia	Total: 3–7	Boys and girls	Hair	Case: 45 Control: 66	Zn: 1.95 ± 0.13 Cu: 1.04 ± 0.1 Fe: 1.11 ± 0.13	Zn: 1.89 ± 0.2 Cu: 1.09 ± 0.21 Fe: 1.1 ± 0.14	8
Avval et al. 2019 [21]	2019	Iran	Asia	Total: 6–12	Boys and girls	Blood	Case: 36 Control: 15	Zn: 85.1 ± 12.59 Fe: 82.9 ± 35.26 Ferritin: 43.4 ± 28.6	Zn: 74.8 ± 15.09 Fe: 73.2 ± 35.9 Ferritin: 37.8 ± 22.91	6
Kaur et al. 2025 [25]	2025	Spain	Europe	Total: 6–15	Boys and girls	Urine	Case: 124 Control: 66	Zn: 428.88 ± 261.24 Cu: 6.57 ± 3.64	Zn: 408.5 ± 325.19 Cu: 3.92 ± 3.99	8
Lukovac et al. 2024 [26]	2024	Serbia	Europe	Total: 6.5–12.5	Boys	Blood	Case: 67 Control: 66	Fe: 16.99 ± 5.69 Ferritin: 40.39 ± 18.67	Fe: 13.75 ± 3.78 Ferritin: 32.72 ± 15.51	7
Mahmoud et al. 2011 [32]	2011	Egypt	Africa	Total: 5–15	Boys and girls	Blood	Case: 58 Control: 25	Zn: 97.5 ± 29.4 Cu: 45.4 ± 26.3 Fe: 24.8 ± 14.1	Zn: 117.4 ± 60.2 Cu: 45.8 ± 23.05 Fe: 32.6 ± 18.7	6
Sakhr et al. 2020 [33]	2020	Egypt	Africa	Total: 6–18	Boys and girls	Blood	Case: 20 Control: 40	Zn: 78.95 ± 16.4 Cu: 207.85 ± 104.88	Zn: 84.14 ± 21.14 Cu: 168.25 ± 87.04	6
Hawari et al. 2020 [34]	2020	Syria	Asia	Total: <12	Boys and girls	Blood	Case: 29 Control: 30	Zn: 83.45 ± 13.42	Zn: 79.97 ± 13.72	7
Bekaroglu et al. 1996 [35]	1996	Turkey	Europe	Total: 6.5–12	Boys and girls	Blood	Case: 48 Control: 45	Zn: 60.6 ± 9.9	Zn: 105.8 ± 13.2	7
Khan 2017 [36]	2017	Saudi Arabia	Asia	Total: 5–12	Boys and girls	Blood	Case: 41 Control: 41	Zn: 0.46 ± 0.15 Fe: 0.95 ± 0.31	Zn: 0.50 ± 0.14 Fe: 1.02 ± 0.29	7
Yorbik et al. 2008 [37]	2008	Turkey	Europe	Total: 7–12	Boys	Blood	Case: 28 Control: 24	Zn: 78.5 ± 15.7	Zn: 107.8 ± 9	6
Perham et al. 2022 [38]	2022	New Zealand	Oceania	Total: 7–12	Boys	Hair	Case: 55 Control: 52	Zn: 168 ± 39 Cu: 31 ± 41 Fe: 13.7 ± 5.6	Zn: 171 ± 46 Cu: 26 ± 21 Fe: 14.3 ± 7.7	7
Arnold et al. 1990 [39]	1990	United States	Americas	Total: 6–12	Boys	Hair Urine	Case: 18 Control: 7	Zn: 163.6 ± 50.2 Zn: 254.8 ± 77.6	Zn: 228.7 ± 167.6 Zn: 363.4 ± 125.1	6
Shin et al. 2014 [40]	2014	South Korea	Asia	Total: 5–15	Boys and girls	Hair	Case: 41 Control: 42	Zn: 12.9 ± 7.26	Zn: 18.69 ± 23.59	7
Zhang et al. 2002 [41]	2002	China	Asia	Total: 6.5–13.5	Boys and girls	Blood	Case: 90 Control: 60	Zn: 12.3 ± 2.3 Cu: 19.9 ± 3.8 Fe: 25.5 ± 5.5	Zn: 13.6 ± 2.1 Cu: 19.9 ± 4.2 Fe: 26.7 ± 5.8	8
Gao et al. 2005 [42]	2005	China	Asia	Total: 6–15	Boys and girls	Blood	Case: 40 Control: 33	Zn: 118.85 ± 20.69 Fe: 10.85 ± 1.51	Zn: 118.24 ± 18.85 Fe: 10.66 ± 1.12	7
Huang et al. 2008 [43]	2008	China	Asia	Total: 6–9	Boys and girls	Blood	Case: 128 Control: 95	Zn: 72.24 ± 7.03 Cu: 14.97 ± 3.51 Fe: 7.64 ± 0.59	Zn: 82.32 ± 9.68 Cu: 14.69 ± 3 Fe: 8.32 ± 0.78	8
Liu et al. 2008 [44]	2008	China	Asia	Total: 6.3–12.5	Boys and girls	Blood	Case: 60 Control: 60	Zn: 63.42 ± 11.9 Fe: 329.15 ± 90.26	Zn: 88.23 ± 12.37 Fe: 346.15 ± 84.24	7
Liu et al. 2006 [45]	2006	China	Asia	Total: 6–14	Boys and girls	Blood	Case: 402 Control: 992	Zn: 0.78 ± 0.17 Cu: 1 ± 0.2	Zn: 0.84 ± 0.18 Cu: 1.13 ± 0.3	8
Zhang et al. 2014 [46]	2014	China	Asia	Total: 1–9	Boys and girls	Blood	Case: 50 Control: 100	Zn: 3.75 ± 0.49 Cu: 5.7 ± 1.33 Fe: 21.33 ± 7.85	Zn: 11.23 ± 4.25 Cu: 6.3 ± 1.58 Fe: 23.57 ± 4.28	7
Asimuguli et al. 2014 [47]	2014	China	Asia	Total: 6–12	Boys and girls	Blood	Case: 150 Control: 150	Zn: 73.95 ± 13.9 Cu: 18.44 ± 4.31 Fe: 8.85 ± 0.7	Zn: 85.99 ± 9.02 Cu: 18.27 ± 4.23 Fe: 8.81 ± 0.77	9
Chen et al. 2006 [48]	2006	China	Asia	Total: 1–10	Boys and girls	Blood	Case: 80 Control: 50	Zn: 90.85 ± 14.47 Cu: 20.36 ± 6.24 Fe: 8.01 ± 0.6	Zn: 93.68 ± 12.28 Cu: 25.6 ± 4.38 Fe: 8 ± 0.67	7
Dong et al. 2014 [49]	2014	China	Asia	Total: 6–7	Boys and girls	Blood	Case: 60 Control: 50	Zn: 73.24 ± 9.899	Zn: 82.51 ± 7.81	7
Huang et al. 2015 [50]	2015	China	Asia	Total: 3–13	Boys and girls	Blood	Case: 65 Control: 60	Zn: 60.12 ± 12.78 Cu: 3.85 ± 0.79 Fe: 62.02 ± 4.58	Zn: 73.98 ± 14.02 Cu: 12.86 ± 3.26 Fe: 82.96 ± 8.12	6
Liu et al. 2003 [51]	2003	China	Asia	Total: 6–10	Boys and girls	Blood	Case: 26 Control: 30	Zn: 4 ± 1.1	Zn: 5.2 ± 1.4	6
Ma et al. 2013 [52]	2013	China	Asia	Total: 1–5	Boys and girls	Blood	Case: 635 Control: 635	Zn: 2.3 ± 0.2 Cu: 0.32 ± 0.04 Fe: 158 ± 30	Zn: 4.3 ± 0.16 Cu: 0.86 ± 0.12 Fe: 362 ± 26.5	9
Mou et al. 2013 [53]	2013	China	Asia	Total: 6–7	Boys and girls	Blood	Case: 70 Control: 60	Zn: 74.39 ± 10.71 Fe: 8.52 ± 0.58	Zn: 80.6 ± 9.07 Fe: 8.76 ± 0.51	8
Tan et al. 2006 [54]	2006	China	Asia	Total: 6.8–13.7	Boys and girls	Blood	Case: 60 Control: 50	Zn: 62.42 ± 12.8	Zn: 73.23 ± 13.36	7
Tan et al. 2008 [55]	2008	China	Asia	Case: 8.32 ± 1.64; Control: 9.02 ± 2.83	Boys and girls	Blood	Case: 36 Control: 35	Zn: 13.444 ± 1.98 Cu: 20.058 ± 3.308	Zn: 13.323 ± 1.718 Cu: 19.229 ± 3.26	6
Zhai et al. 2014 [56]	2014	China	Asia	Total: 0–14	Boys and girls	Blood	Case: 86 Control: 308	Zn: 86.1 ± 30.4 Cu: 18.1 ± 6.1 Fe: 7.9 ± 0.8	Zn: 93.7 ± 23.2 Cu: 17.3 ± 5.4 Fe: 8.1 ± 1.3	8
Liu et al. 2008 [57]	2008	China	Asia	Total: 6.5–13.6	Boys and girls	Blood	Case: 139 Control: 132	Zn: 5.87 ± 1.1 Cu: 6.32 ± 2.14 Fe: 17.44 ± 2.88	Zn: 11.07 ± 2.58 Cu: 6.43 ± 2.16 Fe: 27.03 ± 3.32	8
Tang et al. 2022 [58]	2022	China	Asia	Total: 5–12	Boys and girls	Blood	Case: 1565 Control: 1694	Ferritin: 36.63 ± 20.32	Ferritin: 35.43 ± 20.64	9
Kiddie et al. 2010 [59]	2010	Canada	Americas	Total: 6–12	Boys and girls	Blood	Case: 36 Control: 1581	Zn: 6.6 ± 3.2 Cu: 0.7 ± 0.4 Fe: 17.2 ± 2.34	Zn: 10.0 ± 9.5 Cu: 1.1 ± 1.1 Fe: 14.1 ± 12.9	7
Toren et al. 1996 [60]	1996	Israel	Asia	Total: 6–16	Boys and girls	Blood	Case: 43 Control: 28	Zn: 6.6 ± 3.2	Zn: 10.0 ± 9.5	6
Konofal et al. 2004 [61]	2004	France	Europe	Total: 4–14	Boys and girls	Blood	Case: 53 Control: 27	Ferritin: 23 ± 13	Ferritin: 44 ± 22	7
Konofal et al. 2007 [62]	2007	France	Europe	Total: 5–9	Boys and girls	Blood	Case: 10 Control: 10	Ferritin: 25 ± 15	Ferritin: 46 ± 8	6
Juneja et al. 2010 [63]	2010	India	Asia	Total: 6–14	Boys and girls	Blood	Case: 50 Control: 50	Ferritin: 6 ± 3.9	Ferritin: 49 ± 41.6	6
Menegassi et al. 2010 [64]	2010	Brazil	Americas	Total: 6–15	Boys and girls	Blood	Case: 22 Control: 21	Fe: 78.6 ± 24.0 Ferritin: 54.2 ± 17.2	Fe: 92 ± 31.4 Ferritin: 58.8 ± 28.9	6
Cortese et al. 2012 [65]	2012	France	Europe	Total: 8–14	Boys and girls	Blood	Case: 18 Control: 9	Ferritin: 32.4 ± 13.4	Ferritin: 51.6 ± 16.4	6
Kul et al. 2014 [66]	2014	Turkey	Europe	Case: 9.96 ± 2.48; Control: 9.98 ± 1.94	Boys and girls	Blood	Case: 43 Control: 32	Cu: 17.3 ± 3.2	Cu: 16.9 ± 2.6	7
Abd et al. 2018 [67]	2018	Egypt	Africa	Case: 4.00 ± 2.47; Control: 5.66 ± 3.9	Boys and girls	Blood	Case: 25 Control: 25	Zn: 236.63 ± 20.89	Zn: 144.21 ± 17.4	6

**Acronym:** Zn, Zinc; Cu, Copper; Fe, Iron; NOS, Newcastle-Ottawa Scale.

**Table 2:** Results of the subgroup analysis in this study.

Variables	Number of Studies	SMD (95%CI)	I^2^	*p*-Value
Zn and ADHD				
Age				
≤12 years old	25	−1.25 (−2.05, −0.46)	99.1%	<0.001
6–15 years old	8	−0.64 (−1.19, −0.08)	96.5%	<0.001
Biological sample				
Blood	30	−1.09 (−1.66, −0.52)	99.0%	<0.001
Hair	5	−0.71 (−1.65, 0.22)	94.4%	<0.001
Urine	2	−0.47 (−1.68, 0.74)	83.8%	0.013
Economic level				
Developed countries	7	−0.37 (−0.70, −0.04)	72.1%	0.002
Developing countries	30	−1.14 (−1.73, −0.56)	99.0%	<0.001
Sample size				
<100	15	−0.77 (−1.51, −0.02)	95.8%	<0.001
100–200	13	−0.63 (−1.03, −0.24)	93.5%	<0.001
>200	9	−1.95 (−3.21, −0.69)	99.7%	<0.001
Fe and ADHD				
Age				
≤12 years old	18	−0.66 (−1.48, 0.15)	99.2%	<0.001
6–15 years old	4	−0.91 (−2.48, 0.65)	98.5%	<0.001
Biological sample				
Blood	19	−0.81 (−1.61,− 0.02)	99.2%	<0.001
Hair	4	−0.81 (−2.10, 0.47)	96.1%	<0.001
Economic level				
Developing countries	20	−0.98 (−1.77, −0.19)	99.2%	<0.001
Developed countries	3	0.28 (−0.14, 0.70)	76.4%	0.015
Sample size				
<100	5	−0.74 (−1.89, 0.42)	94.6%	<0.001
100–200	10	−0.36 (−0.84, 0.11)	94.2%	<0.001
>200	8	−1.40 (−2.93, 0.12)	99.7%	<0001
Cu and ADHD				
Age				
≤12 years old	15	−0.66 (−1.58, 0.27)	99.3%	<0.001
6–15 years old	6	0.20 (−0.25, 0.65)	94.6%	<0.001
Biological sample				
Blood	19	−0.66 (−1.32, 0.01)	99.2%	<0.001
Hair	4	−0.28 (−0.66, 0.11)	63.1%	0.044
Urine	1	0.70 (−0.66, 0.11)	-	-
Economic level				
Developing countries	19	−0.74 (−1.41, −0.06)	99.2%	<0.001
Developed countries	5	0.17 (−0.21, 0.55)	81.6%	<0.001
Sample size				
<100	6	−0.46 (−1.05, 0.13)	86.1%	<0.001
100–200	9	−0.24 (−0.87, 0.38)	96.2%	<0.001
>200	9	−0.86 (−1.91, 0.20)	99.6%	<0.001

**Acronym:** Zn, Zinc; Cu, Copper; Fe, Iron; NOS, Newcastle-Ottawa Scale.

## Data Availability

The data presented in this study are available on request from the corresponding author.

## Supplementary Materials

The following supporting information can be downloaded at: https://www.mdpi.com/article/10.3390/nu18111797/s1, Figure S1: Forest plot showing the results of a sensitivity analysis of the pooled estimate of the association between Zn, Fe, ferritin, and Cu levels and ADHD in children and adolescents after excluding any single study. Figure S2: The funnel plot for the test of publication bias of the association between Zn, Fe, ferritin, and Cu levels and ADHD in children and adolescents. Figure S3: Heterogeneity testing for the differences in Zn, Fe, ferritin, and Cu (meta galbraith, random(reml)). Figure S4: Trim and fill adjusted analysis for Zn, Fe, ferritin, and Cu levels with publication bias. Table S1: Summary of literature search strategy. Table S2: Publication bias test in this study. Table S3: Trim and fill adjusted analysis for outcomes with publication bias. Table S4: Investigating potential sources of heterogeneity through meta-regression.
