# Supplementary material for: Essential Trace Elements Zinc, Iron, Copper and Attention-Deficit/Hyperactivity Disorder in Children and Adolescents: A Systematic Review and Meta-Analysis of Case–Control Studies
Source: Nutrients. 2026 Jun 2;18(11):1797. doi: 10.3390/nu18111797 (PMC13258722; doi:10.3390/nu18111797)
Supplement: Supplementary file 1 [file nutrients-18-01797-s001.zip › nutrients-4284761-supplementary.pdf]

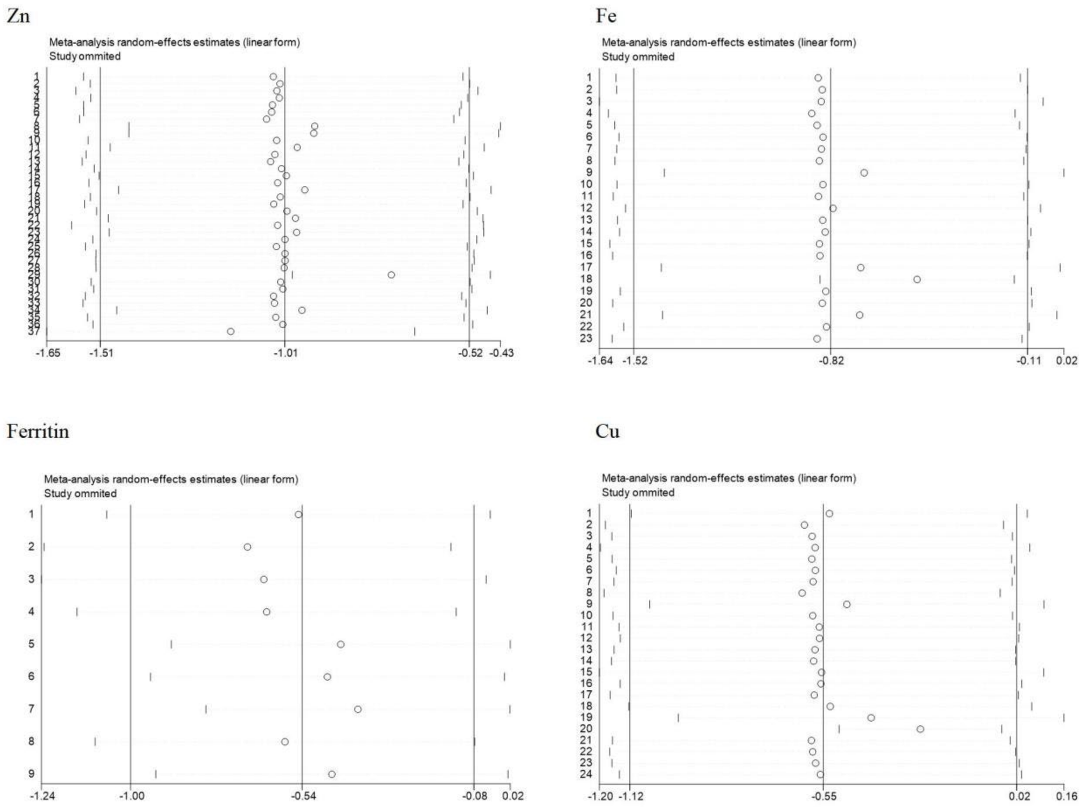

Figure S1. Forest plot showing the results of a sensitivity analysis of the pooled estimate of the association between Zn, Fe, ferritin, and Cu levels and ADHD in children and adolescents after excluding any single study.

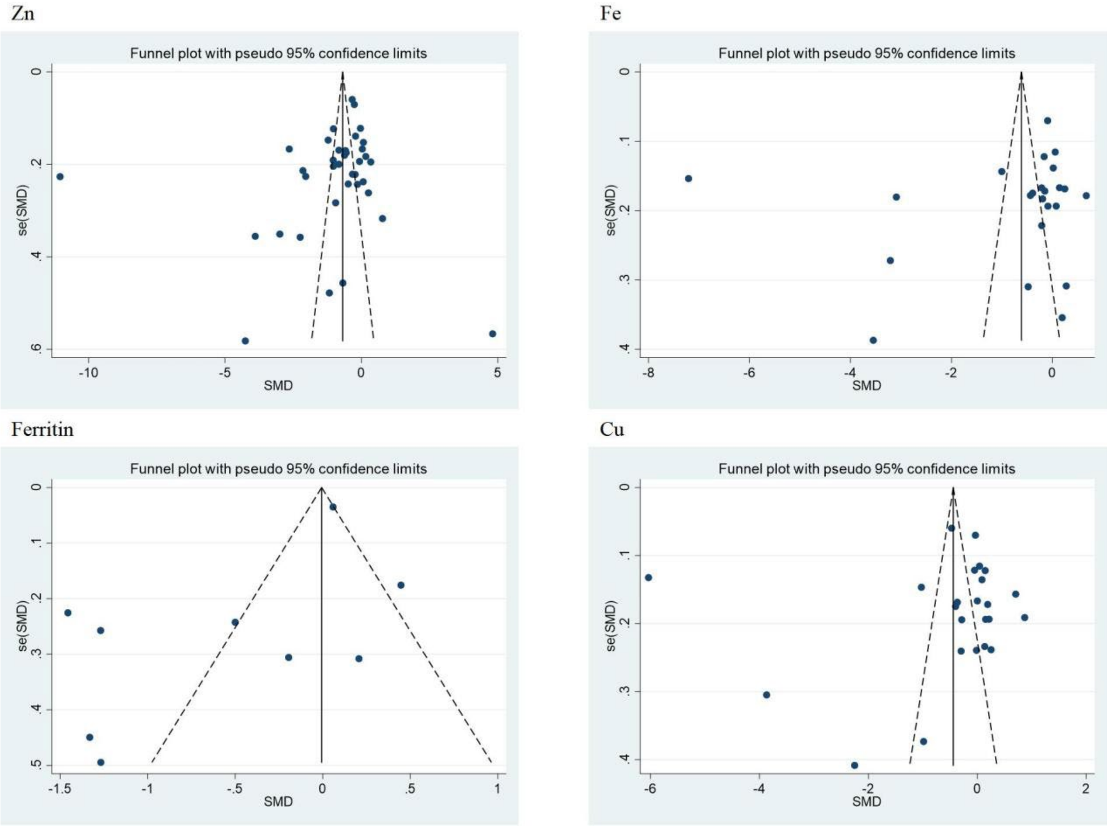

Figure S2. The funnel plot for the test of publication bias of the association between Zn, Fe, ferritin, and Cu levels and ADHD in children and adolescents.

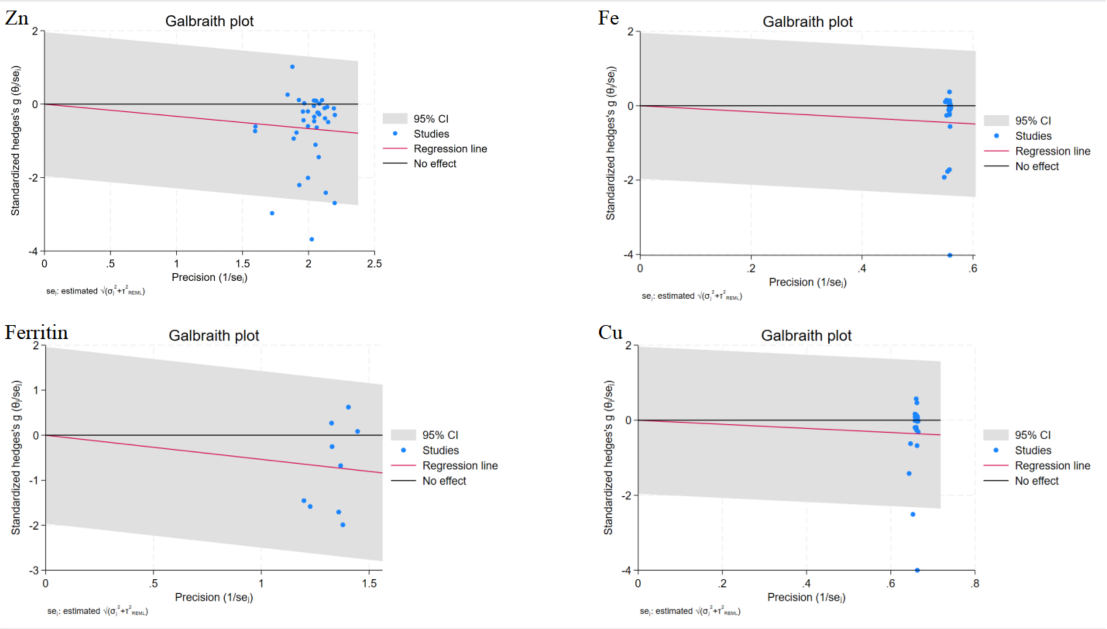

Figure S3. Heterogeneity testing for the differences in Zn, Fe, ferritin, and Cu (meta galbraith, random(reml)).

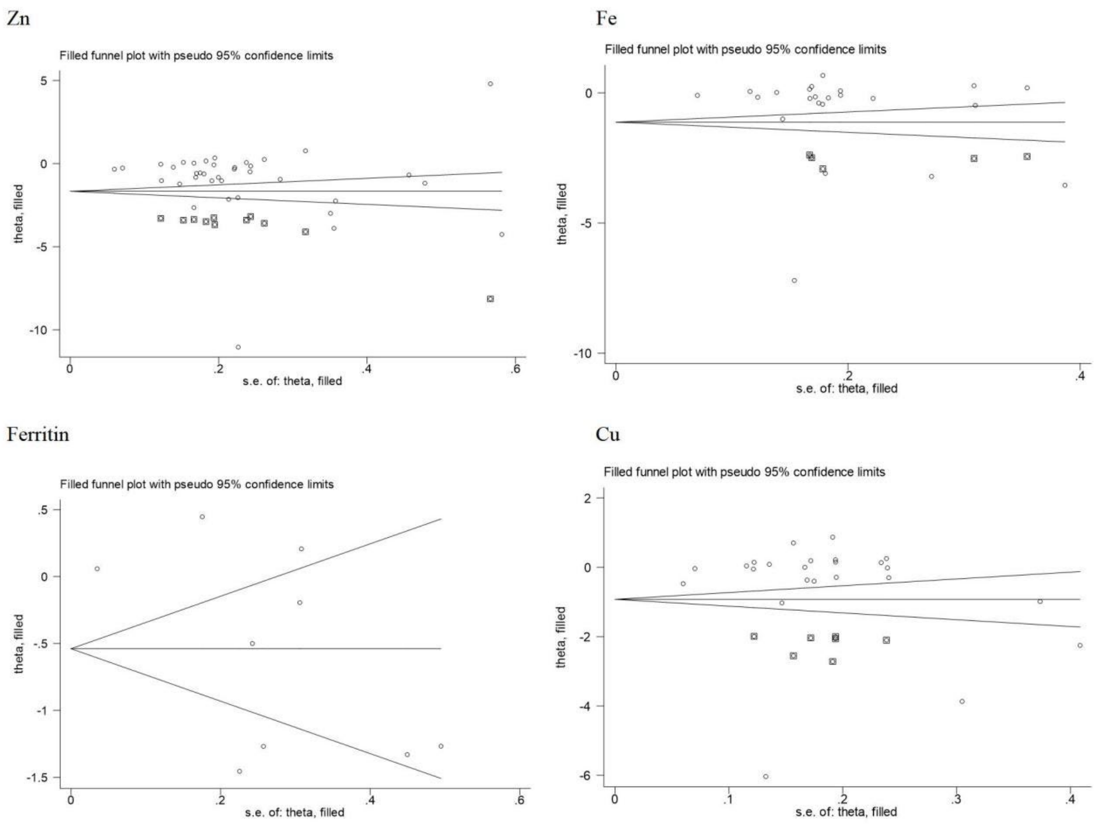

Figure S4. Trim and fill adjusted analysis for Zn, Fe, ferritin, and Cu levels with publication bias.

Table S1. Summary of literature search strategy.

Database	Search date	Limitations	Search terms	Number
Web of Science	March 2026	English language and human studies	(“ADHD” OR “Attention-Deficit/Hyperactivity Disorder” OR “Hyperkinetic Disorder” OR “Conduct Disorder”) AND (“Zn” OR “Fe” OR “Cu” OR “Zinc” OR “Copper” OR “Trace Elements” OR “metal”) AND (“child” OR “adolescent” OR “Teenagers” OR “youth” OR “juvenile” OR “Infant” OR “Young Adult”)	463
Pubmed	March 2026	English language and human studies	(‘ADHD’ OR ‘Attention-Deficit/Hyperactivity Disorder’ OR ‘Hyperkinetic Disorder’ OR ‘Conduct Disorder’) AND (‘Zn’ OR ‘Fe’ OR ‘Cu’ OR ‘Zinc’ OR ‘Copper’ OR ‘Trace Elements’ OR ‘metal’) AND (‘child’ OR ‘adolescent’ OR ‘Teenagers’ OR ‘youth’ OR ‘juvenile’ OR ‘Infant’ OR ‘Young Adult’)	1185
CNKI	March 2026	Chinese language and human studies	Similar Chinese technical terms were adopted to search for eligible articles in CNKI.（主题：ADHD + 多动症 + 注意力缺陷多动障碍 + 多动）AND（主题：儿童 + 青少年 + 婴幼儿）AND（主题：Zn + Cu + Fe + 锌 + 铜 + 铁）	311

Acronym：Zn, Zinc; Cu, Copper; Fe, Iron.

Table S2. Publication bias test in this study.

Variables	Begg's Test	Egger's test
	P-value	P-value
Zn and ADHD	0.065	0.160
Fe and ADHD	0.030	0.445
Ferritin and ADHD	0.466	0.069
Cu and ADHD	0.031	0.792

Acronym：Zn, Zinc; Cu, Copper; Fe, Iron.

Table S3. Trim and fill adjusted analysis for outcomes with publication bias.

Variables	No. of studies	Before adjusted	P-value	No. of studies	After Trim and fill adjusted	P-value	Adjusted studies
Zn and ADHD	37	-1.012(-1.508, -0.516)	<0.001	48	-1.666(-2.168, -1.163)	<0.001	11
Fe and ADHD	23	-0.816(-1.521 ,-0.110)	0.024	28	-1.125(-1.769, -0.481)	0.001	5
Ferritin and ADHD	9	-0.539(-0.999, -0.079)	0.022	9	-0.539(-0.999, -0.079)	0.022	0
Cu and ADHD	24	-0.547(-1.116, 0.021)	0.059	31	-0.924(-1.435,-0.413)	<0.001	7

Acronym：Zn, Zinc; Cu, Copper; Fe, Iron.

Table S4. Investigating potential sources of heterogeneity through meta-regression.

Variables	No. of studies	Regression variables information	Coefficient (95%CI)	P-value
**Zn and ADHD**				
Year of Publication	37	1990-2025	0.009(-0.012,0.030)	0.393
Biological samples	37	1:Urine<br>2:Blood<br>3:Hair	0.008(-0.222,0.238)	0.945
Region	37	1:Europe<br>2:Asia<br>3: Africa<br>4:The Americas	-0.052(-0.286,0.182)	0.664
Sample size	37	25-1617	-0.000(-0.001,0.000)	0.308
**Fe and ADHD**				
Year of Publication	23	2002-225	0.029(-0.098,0.156)	0.653
Biological samples	23	2:Blood<br>3:Hair	-0.008(-1.200,1.983)	0.994
Region	23	1:Europe<br>2:Asia<br>4:The Americas<br>5: Oceania	0.262(-0.620,1.144)	0.561
Sample size	23	36-1617	-0.001(-0.003,0.000)	0.108
**Ferritin and ADHD**	-	-	-	-
**Cu and ADHD**				
Year of Publication	24	2002-2025	0.017(-0.089,0.123)	0.756
Biological samples	24	1:Urine<br>2:Blood<br>3:Hair	-0.099(-1.514,1.317)	0.891
Region	24	1:Europe<br>2:Asia<br>3: Africa<br>4:The Americas<br>5: Oceania	-0.032(-0.723,0.658)	0.927
Sample size	24	36-1617	-0.001(-0.002,0.000)	0.108

Acronym：Zn, Zinc; Cu, Copper; Fe, Iron.
